# Parallel action of AtDRB2 and RdDM in the control of transposable element expression

**DOI:** 10.1186/s12870-015-0455-z

**Published:** 2015-03-03

**Authors:** Marion Clavel, Thierry Pélissier, Julie Descombin, Viviane Jean, Claire Picart, Cyril Charbonel, Julio Saez-Vásquez, Cécile Bousquet-Antonelli, Jean-Marc Deragon

**Affiliations:** Université de Perpignan Via Domitia, LGDP UMR CNRS-UPVD 5096, 58 Av. Paul Alduy, 66860 Perpignan Cedex, France; CNRS UMR5096 LGDP, Perpignan Cedex, France; Present address: IBMP, UPR 2357, 12, rue du général Zimmer, 67084 Strasbourg cedex, France; Present address: UMR6293 CNRS - INSERM U1103 – GreD, Clermont Université, 24 avenue des Landais, B.P. 80026, 63171 Aubière Cedex, France

**Keywords:** RNAi, siRNA, Double-stranded RNA binding protein, Epigenetics, Chromatin, Arabidopsis

## Abstract

**Background:**

In plants and animals, a large number of double-stranded RNA binding proteins (DRBs) have been shown to act as non-catalytic cofactors of DICERs and to participate in the biogenesis of small RNAs involved in RNA silencing. We have previously shown that the loss of *Arabidopsis thaliana*’s DRB2 protein results in a significant increase in the population of RNA polymerase IV (p4) dependent siRNAs, which are involved in the RNA-directed DNA methylation (RdDM) process.

**Results:**

Surprisingly, despite this observation, we show in this work that DRB2 is part of a high molecular weight complex that does not involve RdDM actors but several chromatin regulator proteins, such as MSI4, PRMT4B and HDA19. We show that DRB2 can bind transposable element (TE) transcripts *in vivo* but that *drb2* mutants do not have a significant variation in TE DNA methylation.

**Conclusion:**

We propose that DRB2 is part of a repressive epigenetic regulator complex involved in a negative feedback loop, adjusting epigenetic state to transcription level at TE loci, in parallel of the RdDM pathway. Loss of DRB2 would mainly result in an increased production of TE transcripts, readily converted in p4-siRNAs by the RdDM machinery.

**Electronic supplementary material:**

The online version of this article (doi:10.1186/s12870-015-0455-z) contains supplementary material, which is available to authorized users.

## Background

RNA recognition by proteins is based on a number of specialized amino acid modules that interact with the structure and/or the primary sequence of their RNA targets. The double-stranded RNA binding motif (DSRM) is an example of such module. The DSRM is an evolutionary conserved 65 to 68 amino acids region that can adopt a typical α − β − β − β − α fold with the most conserved residues mainly located in its C-terminal part [1-3]. DSRMs bind perfect or imperfect RNA-RNA duplexes (but not RNA-DNA or DNA-DNA duplexes) by contacting two ribose 2′-OH residues on each side of the sugar backbone [2,4,5]. Consequently, DSRM-containing proteins bind RNA based essentially on structural features and not on primary sequences although, in some cases, a specific primary sequence can influence binding by inducing a particular RNA secondary structure [6,7]. DSRM are often found in multiple copies and/or associated with other functional domains such as Ribonuclease III, DEAD/DEAH box helicase, PAZ, serine/threonine kinase, phosphatase and adenosine deaminase (for a review see [2]). DSRM-containing proteins have been involved in a number of biological functions including cellular mRNA transport and localization [8,9], RNA maturation [10-13], mRNA edition [14] and degradation [15-18], mRNA translation [19-21] and RNA interference processes [22-27].

In plants, DSRM-containing proteins have been essentially involved in the RNA interference process. Eighteen DSRM proteins are present in *Arabidopsis thaliana*, including four Dicer-Like (DCL) and five double-stranded RNA binding (DRBs) proteins [28-30]. DCLs are key enzymes involved in the biogenesis of the different classes of small interfering RNAs and are composed of one or two DSRM associated with RNase III, PAZ, DUF283 and helicase domains. DCL1 is responsible for the production of 21 nucleotides microRNAs from RNA polymerase II precursor transcripts [31] as well as for the production of phased *cis* natural antisens siRNAs, while DCL2 cleaves the primary convergent transcripts into 24 nucleotides duplex in this pathway [32]. DCL2 is also implicated in gene silencing induced by exogenous dsRNAs, as is DCL4 [33,34]. DCL4 also generates phased trans-acting siRNAs from dsRNA provided by the action of a miRNA loaded RISC and RNA-dependent RNA polymerase 6 (RDR6) [35] and is also responsible for the formation of some microRNAs [36]. Finally, DCL3 acts in the RNA-dependent DNA methylation (RdDM) pathway on precursor molecules generated by RNA polymerase IV and RNA-dependent RNA polymerase 2 (RDR2), to produce essentially 24 nucleotides p4-siRNAs that guide DNA methylation, mostly to repeated sequences and transposable elements, thus participating in genome defense [37-39]. Other major actors of the RdDM pathway include Argonaute 4 (AGO4) and RNA polymerase V, both involved in the recruitment of DNA methylation enzymes [38,39].

Plant DRBs are strictly composed of two DSRMs with no other functional domain. *Arabidopsis* possesses five known DRB (DRB1 to 5) [29], each containing two N-terminal DSRMs. DRB1 and DRB4 have been well characterized and act as non-catalytic cofactors of DCLs. DRB1, also known as HYL1, is required for DCL1-mediated processing of miRNA precursors [40]. DRB1 acts as a dimer and interact with DCL1 via its second DSRM [41,42], while the first DSRM binds miRNA precursors as well as mature miRNA duplexes [43,44], assisting in the cleavage and in the miRNA strand selection. DCL4 is assisted by DRB4 [45] and this protein is essential for DCL4 *in vitro* activity [46]. DRB4 has also a role in resistance against pathogens, distinct from its action alongside DCL4 [47]. The role of the three other *Arabidopsis* DRBs is more elusive. DRB3 seems to interact with DCL3, impacting the methylation of a viral genome [48] while DRB2, DRB3 and DRB5 have all been implicated in an atypical miRNA biogenesis pathway [49]. In a previous work, we have shown that mutants deficient in DRB2 accumulate higher amounts of p4-siRNAs [50], suggesting a role for this protein in the RdDM pathway. In this work, we demonstrate that DRB2 is part of a high molecular weight nuclear complex containing many co-repressors and chromatin regulatory factors, suggesting that changes in p4-siRNA levels in *drb2* mutant may be the consequence of uncontrolled transcription of RdDM loci. We proposed that the binding of nascent transcripts by DRB2 might facilitate the recruitment of repressing epigenetic factors that provide fine-tuning of transcription at targeted loci. Loss of DRB2 would mainly result in an increased production of transposable element transcripts that would be readily converted in p4-siRNAs by the RdDM machinery.

## Results

### *In vivo*, DRB2 exists as a nuclear high molecular weight complex

**Figure 1:**
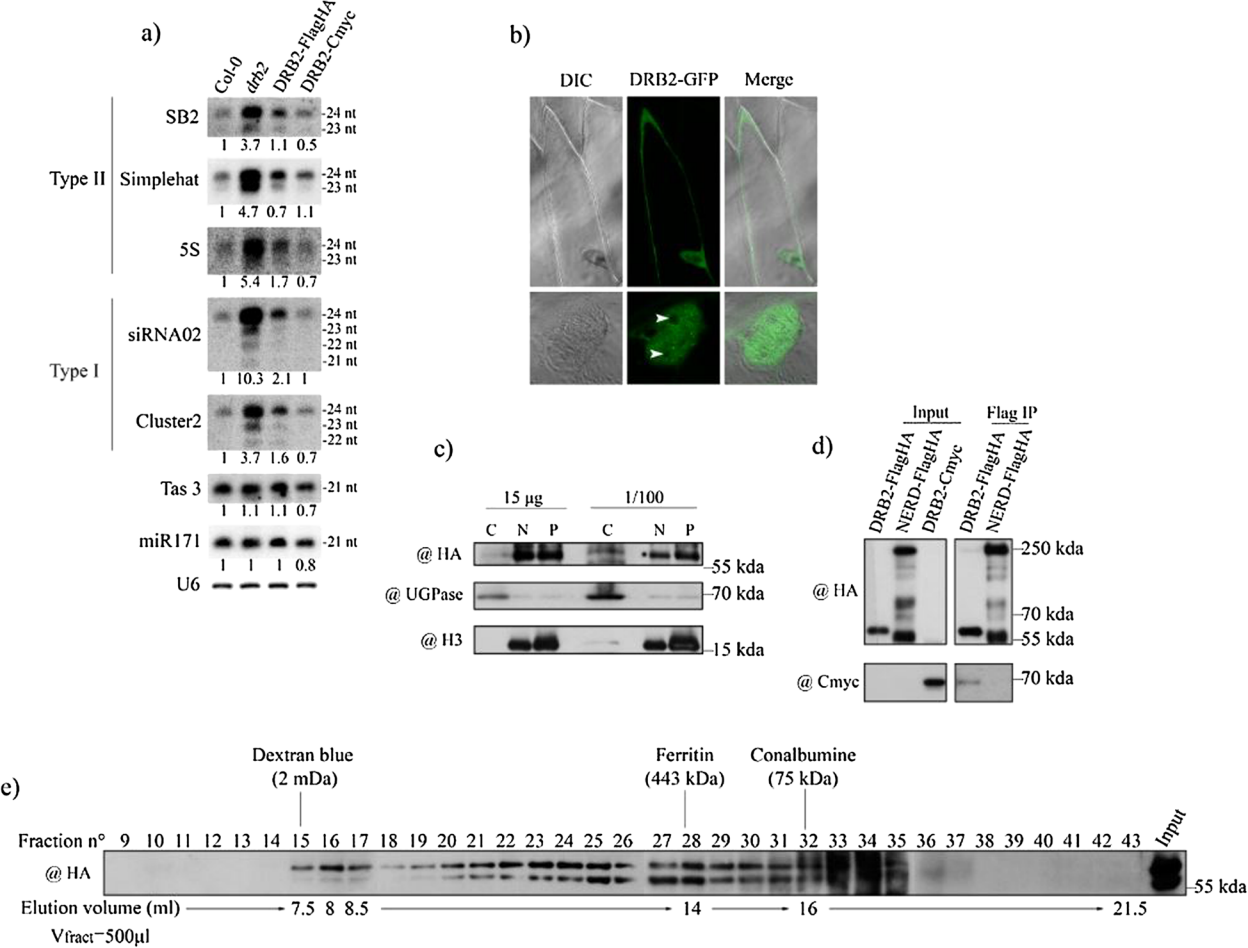
**DRB2 is found predominantly in the nucleus and forms a high molecular weight complex as well as a homo interaction. (a)** Level of small RNA accumulation in wild-type (Col-0), *drb2-1* and two complementing lines. Values are normalized to U6 RNA and are expressed as a ratio relative to Col-0. For p4-siRNAs, only the 24-nt species were used for normalization. **(b)** Subcellular localization of DRB2-GFP in a heterologous system. GFP signal is observed both in the cytoplasm and the nucleus, but is absent from the nucleolus. **(c)** Subcellular localization of DRB2-FlagHA by cell fractionation and western blot. DRB2-FlagHA appears to be mainly nuclear. Extracts from each compartment were loaded in a SDS-PAGE either as a fixed protein quantity (first three lanes) or as 1/100th of the total extract (last three lanes). C stands for cytoplasm, N for nucleus and P for pellet. DRB2-FlagHA is revealed with a commercial HA antibody (@), UGPase is used as the cytosol quality control and H3 as the nuclear quality control. **(d)** Coimmunopurification of the DRB2-Cmyc protein from DRB2-FlagHA bound Flag magnetic beads. DRB2-FlagHA is able to bind DRB2-Cmyc, while NERD-FlagHA is not. DRB2-FlagHA and NERD-FlagHA are both revealed with a commercial HA antibody and the presence of DRB2-Cmyc in the DRB2-FlagHA eluate is revealed with a Cmyc commercial antibody. **(e)** Gel filtration on a Superose 6 column of DRB2-FlagHA crude extracts. The elution profile of DRB2-FlagHA shows that it is present in a high molecular weight complex of an approximate mass of 2 MDa as well as in the intermediate forms of lower mass of this complex. Fractions (500 μl) were analysed by western blot, and DRB2-FlagHA is revealed with HA antibody. Fraction numbers, sizing standards and corresponding volumes are indicated.

Since the *drb2* mutation leads to an increase in the abundance of p4-siRNA of all sizes (21-nt to 24-nt) and classes (Type I and II) [50], we set out to document the role of *DRB2* in the RdDM pathway. As a first step, we generated transgenic plant lines in the *drb2-1* background, expressing the complete *DRB2* genomic sequence, under the control of its own promoter, defined as the whole intergenic region (3.4 kb) upstream of *DRB2*, fused in C-terminal to either two Flag and two HA tags (FlagHA), or four Cmyc tags (Cmyc). Figure 1a shows that first generation transformed plants restore a wild-type like accumulation of p4-siRNAs (compare the Col-0 lane to the DRB2-FlagHA and DRB2-Cmyc lanes), in contrast to the symptomatic over accumulation phenotype of *drb2* (*drb*2 lane), while they do not affect the quantity of both Tas3 and miR171 small RNAs. Homozygous descendants from these plants were considered as complemented lines and were used in the following studies. In order to document the subcellular localization of DRB2, the above-mentioned *DRB2* genomic construct was fused to the coding sequence of GFP (Green fluorescent protein) and bombarded into onion cells. In all observed cells, DRB2 was found in the cytoplasm and in the nucleoplasm, while consistently excluded from the nucleolus (Figure 1b). Although a GFP signal of similar intensity is present in both cytoplasm and nucleoplasm, DRB2-FlagHA appears to be mainly nuclear when cell fractionation is performed (Figure 1c). Whether a fixed quantity of protein or a fixed proportion of each extract is analysed, DRB2 is found mainly in the total nuclear extract (“N” lanes) as well as in the remaining insoluble nuclear pellet (“P” lanes). The DRB2-FlagHA signal observed in the cytoplasmic fraction is weak (“C” lanes), but likely significant as DRB2 can be immunoprecipitated from cytoplasmic extracts (data not shown). Altogether, these data show that DRB2 is enriched in the nucleus, suggesting that its main function occurs in this compartment.

Knowing that HYL1/DRB1 binds miRNA/miRNA* duplexes as a homodimer [41], we tested if two DRB2 molecules could interact *in planta*. We immunoprecipitated DRB2-FlagHA using anti Flag magnetic beads, which were then challenged with DRB2-Cmyc extracts. A specific signal is obtained for DRB2-cmyc in the DRB2-FlagHA IP, but not in the NERD-FlagHA negative control [51], which is derived from the same plasmid (Figure 1d). This suggests that DRB2 can indeed make homo interactions *in vivo*. As four other DRB proteins exist in *Arabidopsis*, we also tested if DRB2 is able to interact with other DRBs, especially DRB4, as the *drb4* mutation shows an opposite molecular phenotype to that of *drb2* [50]. In cotransformed plants possessing both the DRB2-FlagHA and the DRB4-Cmyc constructs, no Cmyc signal is observed after a Flag IP (Additional file 1: Figure S1a). Similarly, no signal was observed after a Flag IP for DRB1 and DRB5 using custom made antibodies (Additional file 1: Figure S1a).

To document the possible ability for DRB2 to form further complexes, we performed size fractionation experiments. When eluted through a Superose 6 column, which allows good separation of high molecular weight material, DRB2-FlagHA is found in a peak near the 2 MDa molecular marker (Figure 1e, fractions 15 to 17) and throughout the following fractions down to fraction 35. This result suggests DRB2 is part of a high molecular weight complex with a maximal approximate size of 2 MDa and that the signal observed in fractions 18 to 35 reflects intermediate forms of this complex down to the monomeric form. We were able to stabilize this high molecular weight complex by incubating for only five minutes with a crosslinking agent (dithiobis[succinimidylpropionate], DSP), although an important proportion of DRB2-FlagHA still remains as a monomer (the signal between 55 kDa and 70 kDa) (Additional file 1: Figure S1b). Lower molecular weight intermediate forms could also be observed by lowering the concentration of DSP (Additional file 1: Figure S1b). As the maximum elution size of DRB2-FlagHA is close to that of the dextran and might be excluded from the column (Figure 1e), we performed the size fractionation experiment again, and collected smaller fractions (250 μl). This way, we were able to see that DRB2-FlagHA is included in the resolving range of the column (Additional file 1: Figure S1c).

Altogether, our data suggests that DRB2 is present in a high molecular weight complex of approximately 2 MDa that probably function in the nucleus, and that although DRB2 is likely able to form a dimer, it does not interact with the other tested DRBs.

### The DRB2 complex is devoid of the major components of the RdDM pathway

Since DCL1 and DCL4 respectively necessitate DRB1 and DRB4 to achieve proper small RNA production [40,45], we decided to test if DRB2 is also a DCL cofactor. Using the DRB2-FlagHA complemented line and antibodies raised against DCL1, DCL3 and DCL4, we performed Flag IPs using an experimental set up very similar to the one we used previously to detect the DRB4/DCL4 interaction *in vivo* [50], but could not detect any interactions (Additional file 2: Figure S2a). The same approach was also used to test the interaction to other main components of RdDM, namely polymerases IV and V, RDR2 and AGO4. Additional file 2: Figures S2b, S2c and S2d show that, as it is the case for DCLs, no interaction could be seen between DRB2 and RDR2, AGO4, NRPD1 and NRPE1 (which correspond respectively to the largest subunit of Polymerase IV and V [52,53]). Taken together, these results suggest that DRB2 does not interact with any of these RdDM components, and that the p4-siRNA accumulation phenotype of the *drb2* mutant (Figure 1a) is likely not linked to a direct role for DRB2 in this particular pathway.

We next tested the impact of the *drb2* mutation on DNA methylation. In accordance with the observed over accumulation of p4-siRNA (Figure 1a), one could expect hypermethylation of transposable elements (TEs) loci controlled by these p4-siRNAs. DNA methylation levels were assessed by bisulfite sequencing for non-autonomous short interspersed element (SINEs) individual copies that have numerous p4-siRNAs matching their genomic sequence. No significant variation is obtained for SB2-2, SB3-35 and AtSN1 (SB4-8) SINE copies in the *drb2* mutant (Additional file 2: Figures S2e, f and g), while the *nrpe1* mutation, included here as a control, clearly affects CHG and CHH methylation as well as CG methylation to some extent.

We also investigated the possibility that the *drb2* mutation results in changes in TE RNA levels. No reproducible change in RNA accumulation was observed for a diverse set of TEs in the *drb2* background compared to the wild type situation (data not shown). We also generated *drb2* x *ddm1* lines, taking advantage of the *ddm1* background, known to accumulate several TE transcripts [54-56]. We observed that despite the higher level of p4-siRNAs linked to the *drb2* mutation (Figure 1a), changes in steady state levels of TE RNAs are weak and not always significant in the *drb2/ddm1* double mutant compared to the single *ddm1* mutant (Additional file 3: Figure S3).

Overall, these results suggest that DRB2 does not play a major role in maintaining correct methylation pattern in RdDM and that the *drb2* mutation is not associated with significant modifications of steady-state levels of full length TE RNAs.

### DRB2 interacts with many proteins linked with chromatin regulatory functions

To further investigate the role of DRB2, we performed affinity purification from floral tissues of the DRB2-Cmyc line and mass spectrometry was used to reveal co-purifying proteins. IPs from DRB2-Cmyc and Col-0 were subjected to SDS-PAGE and specific bands appearing in the DRB2-Cmyc lane were cut and analysed separately. After removing contaminants found in the Col-0 extract, DRB2 was found to be the top scoring protein at its expected size, with a good coverage and emPAI (Table 1). Interestingly, many of the co-purifying proteins have previously been described as epigenetic regulators. PRMT4B (PROTEIN ARGININE METHYLTRANSFERASE 4B) is able to methylate numerous arginines from the H3 histone and has been implicated in the regulation of flowering time [57]. Similarly, HDA19/HD1 (HISTONE DEACETYLASE 19/1) acts directly on chromatin by removing acetyl groups from various H3 and H4 lysines and has been implicated in a wealth of biological processes [58-60], notably apical embryonic fate [61] and floral identity [62] alongside TPL (TOPLESS) which is also found in the IP. Also found in the DRB2-Cmyc affinity purification are: NFA03 (NUCLEOSOME ASSEMBLY PROTEIN 03) a protein homologous to an animal histone chaperone, two chromatin remodelling factors, NUC1 (NUCLEOLIN-LIKE1) and SWI3A (SWITCH3A) [63,64], MBD10 a protein involved in the recognition of methylated cytosines, and implicated in nucleolar dominance in the hybrid species *A. suecica* [65]. Surprisingly, AGO4 is found in two different bands although we have previously been unable to document an interaction with DRB2 (Additional file 2: Figure S2b).

**Table 1:** **Mass spectrometric analysis of DRB2-Cmyc affinity purification**

**In gel**	**Protein**	**AGI code**	**Score**	**Coverage (%)**	**Unique peptides**	**SC**	**emPAI**
**55 < 72**	DRB2	At2g28380	1372.8	59.2	18	31	6.32
	PRMT4B	At3g06930	668.1	26.9	9	11	0.76
	MSI4	At2g19520	532.8	28.3	8	10	0.73
	WD40 containing	At3g18060	370.1	20.4	8	8	0.45
	NUCL1	At1g48920	358.8	12	6	6	0.37
	HDA19	At4g38130	339.7	11.2	4	4	0.24
	SWI3A	At2g47620	200	7.9	3	3	0.17
	MBD10	At1G15340	195.3	15.6	5	5	0.43
	WD40 containing	At5g24710	115.1	1.4	2	2	0.04
	WD40 containing	At2g01330	87.7	6	2	2	0.13
**<55**	NFA03	At5g56950	287.5	12.7	5	5	0.42
	PRL1	At4g15900	228.5	15.7	5	5	0.33
	AKIN10	At3g01090	103.1	6.6	3	3	0.17
	WD40 containing	At1g04510	82.1	4.7	2	2	0.11
**95 < 130**	AGO4	At2g27040	172.6	4.1	4	4	0.13
**>250**	ACC1	At1g36160	1959.2	23	43	43	0.7
	WD40 containing	At2g21390	568.5	10.3	11	11	0.28
	AGO4	At2g27040	167.1	4.7	4	4	0.13
	CAND1	At2g02560	117.8	3.1	3	3	0.07
	TPL	At1g15750	113.2	4.7	5	5	0.13

Table summarizing the multiple proteins specifically found in the DRB2-CMyc purified extract. Proteins found in one cut band are grouped, and all are ordered by their respective scores. Protein names as well as the corresponding AGI codes, the score of each protein, the percent coverage for the known protein sequence and the number of unique peptides matching to the protein are given. The spectral count (SC) is the total number of sequenced peptides for a protein, and the exponentially modified protein abundance index (emPAI) (defined as 10^Nobserved/Nobservable^ – 1, were N is either the number of observed peptides or the number of theoretically observed peptide after trypsin digestion), is indicated to estimate the abundance of a given protein in an extract [66].

One of the top scoring proteins in our mass spectrometry analysis, MSI4 (MULTICOPY SUPPRESSOR OF IRA1 4), has also been implicated in the epigenetic regulation of flowering time and cold response [67,68] as well as in the transcriptional control of TEs [69,70]. MSI4 also acts as a substrate adaptor in CUL4-DDB1 ubiquitin E3 ligases via its WDxR motif, and it has been shown that the CUL4-DDB1^MSI4^ complex is present at FLC chromatin and interacts with a component of the polycomb repressive complex 2 (PRC2) [71], thus regulating flowering. Intriguingly many other DCAFs (DDB1-CUL4 ASSOCIATED FACTORS), which contain the WDxR motif inside a WD40 domain [72], are also purified alongside DRB2. This is the case for MSI4, PRL1 (PLEIOTROPIC REGULATORY LOCUS 1), At3g18060 and At2g01330, while the IP contains other WD40 containing proteins not classified as DCAFs (TPL, At5g24710, At1g04510, At3g63460, at2g21390). Accordingly, the IP also contains AKIN10, a Snf1-related protein kinase, which is ubiquitylated by the CUL4-DDB1^PRL1^ complex to promote its degradation [72], and CAND1 (CULLIN ASSOCIATED AND NEDDYLATION DISSOCIATED), a protein acting as an inhibitor towards CUL4 [73].

**Figure 2:**
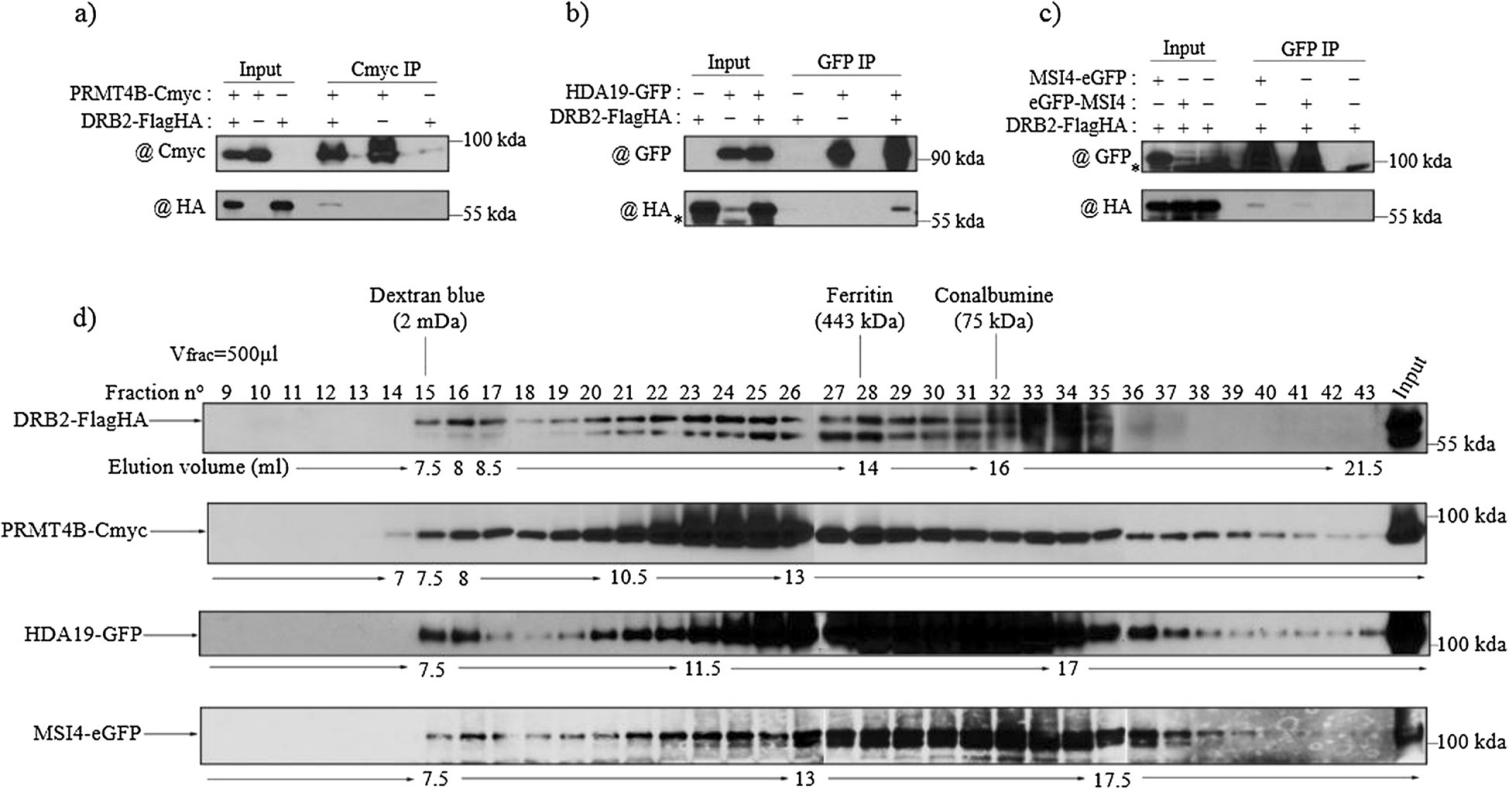
**DRB2 interacts** ***in planta*** **with proteins involved in chromatin regulation. (a-c)** Co-immunoprecipitations confirming the interaction between DRB2 and **(a)** PRMT4B, **(b)** HDA19, and **(c)** MSI4. For each experiment, F1 plants resulting from the cross between the two lines and harbouring both transgenes were used, while either the parental line or a F1 plant segregating only one of the transgenes were used as negative controls. Inputs and purified fractions were analysed by western blot. Background bands are indicated by an asterisk (*). **(d)** Gel filtration on a superose 6 column of DRB2-FlagHA, PRMT4B-Cmyc, HDA19-GFP and MSI4-eGFP crude extracts. Fractions (500 μl) were analysed by western blot and fraction numbers, sizing standards and corresponding volumes are indicated. In all cases, DRB2-FlagHA is revealed with a HA antibody, PRMT4B-Cmyc with a Cmyc antibody and HDA19-GFP, MSI4-eGFP, eGFP-MSI4 are revealed using a GFP antibody.

In order to confirm the interaction between some of these proteins and DRB2, *in planta* co-immunoprecipitations were performed using the DRB2-FlagHA line and epitope tagged versions of PRMT4B, MSI4, HDA19 and MBD10 (the interaction with AGO4 having been tested previously, see Additional file 2: Figure S2b). Consistent with the MS analysis, we are able to observe co-IP of DRB2-FlagHA with PRMT4B-Cmyc after a Cmyc IP (Figure 2a), with HDA19-GFP after a GFP IP (Figure 2b) in F1 plants possessing both epitope tagged proteins. Similarly, a specific signal for DRB2-FlagHA is obtained after a GFP IP with both MSI4-eGFP and eGFP-MSI4 proteins, while no signal is observed when only DRB2-FlagHA is present in the crude extract (Figure 2c). This interaction is also observed when both proteins are transiently expressed in *Nicotiana benthamiana* (Additional file 4: Figure S4). However, using a similar IP protocol on extracts from plants co-expressing DRB2-FlagHA and MBD10-Cmyc, we were not able to confirm *in planta* the DRB2-MBD10 interaction suggested by the mass spectrometry data (not shown).

To further characterize the complex formed by DRB2 and its partners, we performed gel filtration on the same Superose 6 column for all the epitope tagged interacting proteins. Consistent with an interaction with DRB2, PRMT4B-Cmyc, HDA19-GFP and MSI4-eGFP all elute in the same maximal fraction, around 2 MDa, as it is the case for DRB2-FlagHA, although their elution profiles are not strictly identical (Figure 2d). PRMT4B has the profile resembling the most that of DRB2, with enrichment infractions 21 to 26, while HDA19-GFP and MSI4-GFP show a broad peak between fractions 20 and 33, and fractions 26 to 33 respectively (Figure 2d). Nevertheless, our results support the notion that these proteins form a large multimeric complex, at a maximal size around 2 MDa.

### DRB2 is able to bind transposable element transcripts in *vivo*

DRB2 harbours two N-terminal DSRMs whose presence is conserved in all *A. thaliana*’s DRBs and allows DRB1/HYL1 and DRB4 to bind to double-stranded RNA [41,46]. *In vitro* reconstituted DRB2 is able to strongly bind to a perfect double-stranded RNA substrate [29] suggesting the existence of *in vivo* RNA targets for this protein. We first assayed the binding of DRB2-FlagHA to small RNA duplexes by IP and subsequent labelling with [5′ ^32^P]pCp (cytidine-3′,5′-bis-phosphate), which allows detection of any kind of RNA with free 3′-OH moieties. No specific signal was obtained for abundant cellular RNAs between 100-nt and 70-nt (Additional file 5: Figure S5a) and more importantly, no enrichment was observed for small RNAs between 30-nt and 20-nt (Additional file 5: Figure S5b) suggesting that unlike DRB1/HYL1, DRB2 is unable to bind small RNA duplexes.

**Figure 3:**
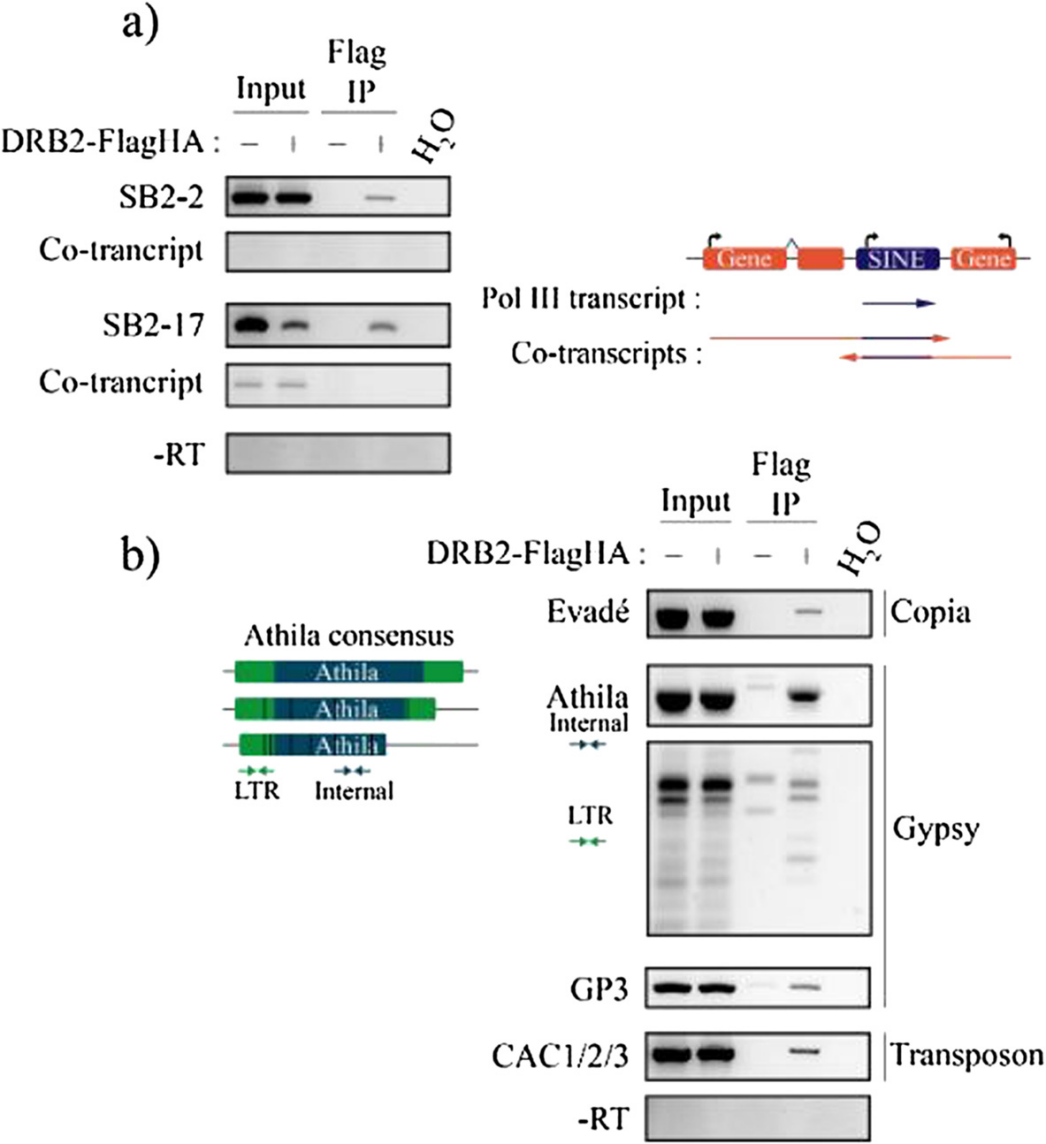
**DRB2 is able to bind TE transcripts. (a)** RNA Immunoprecipitation (RIP) from mixed floral tissues of SINE transcripts in DRB2-FlagHA x *ddm1* plants and *ddm1* plants included as a negative control. Total RNA is extracted following the IP, DNase treated and reverse transcribed. PCR amplification is performed with primers specific to one element, and a second set of primers specific to the putative co-transcript. Each time, a control reaction is performed with water instead of matrix cDNA (H_2_O), and each time, absence of contaminant genomic DNA is assessed by performing the same amplification with the non-reverse transcribed material (−RT). **(b)** Same RIP experiment performed on a diverse set of TEs, one Copia, two Gypsies and one CACTA. Primer sets used to amplify the Athila family are designed on a consensus sequence and can therefore amplify numerous genomic copies, both in the LTR and in the internal sequence. Evadé, GP3 are locus specific primers while CAC1/2/3 primers detect three different loci. The same control reactions are performed.

We next asked whether DRB2 is able to bind transcripts arising from TEs, which would be consistent with its presence in a chromatin regulatory complex. IPs followed by RT-PCR were performed in the *ddm1* mutant, allowing for easier detection of low abundance TE transcripts. Specific signal was obtained for the DRB2-FlagHA x *ddm1* IP for both SB2-2 and SB2-17 TE transcripts (Figure 3a). As SINEs tend to be inserted close to genes in euchromatic regions [74,75], amplifications with primers around the SINEs were used to detect possible co-transcripts. Although a low level of co-transcript was observed for SB2-17 in the input, no such transcripts were seen in the IP, suggesting that DRB2 is able to bind efficiently highly structured SINE transcripts [76] originating from Pol III transcription (Figure 3a). Transcripts from a diverse set of TEs were assayed in the same fashion and yielded similar results (Figure 3b). Evadé, a LTR retrotransposon from the Copia family, prone to transcriptional reactivation in *met1* and *ddm1*, is also found in the *ddm1* x DRB2-FlagHA IP (Figure 3b) but not in the Col-0 IP (Additional file 5: Figure S5c). The detection of Athila transcripts was achieved with primers matching numerous copies of this abundant Gypsy class TE, and specific signals were obtained with both the LTR and the internal sequence. Transcripts from GP3, another Gypsy element presenting new genomic insertions in self-pollinated *ddm1* plants [56] were also found bound to DRB2-FlagHA, as were CAC1/2/3 (Cacta) and Vandal 21 (MuDR) transcripts, both DNA transposons (Figure 3b and Additional file 5: Figure S5c).

## Discussion

Animal DRBs have been involved in many different functions [8,9,15,19,21,22,25-27], but surprisingly this is not the case for plant DRBs that so far have been strictly associated with the biogenesis of small RNAs in diverse RNA interference processes [29,40-42,44,46-50,77]. *Arabidopsis thaliana drb2* mutants present a 2 to 10 fold increase in RdDM associated p4-siRNA ([50] and Figure 1a) again suggesting that plant DRB2 is involved in regulating small RNA biogenesis and is therefore a regulator of RdDM. However, we show in this work that DRB2 influences p4-siRNA accumulation in a process that likely works independently of the RdDM pathway.

We first observed that DRB2 is mainly a nuclear protein, that can possibly form a homodimer and can further associates to other proteins to form a high molecular weight complex *in vivo* (Figure 1). Based on the molecular phenotype of *drb2* plants (an increase in p4-siRNAs), we initially developed a targeted approach to identify DRB2 partners among RdDM actors, starting with DCL3, the enzyme involved in cutting p4-siRNA precursors [39]. With an experimental set up very similar to the one we used to detect the DRB4/DCL4 interaction *in vivo* [50], we were unable to show that DRB2 interact with DCL3, or with any of the other DCLs tested (Additional file 2: Figure S2). This result suggests that, in contrast with DRB1 and DRB4, DRB2 likely isn’t a DCL cofactor. We also observed that DRB2 does not interact with other major RdDM actors (PolIV, PolV, RDR2 and AGO4, Additional file 2: Figure S2). In addition, *drb2* plants, despite presenting a high level of p4-siRNAs, do not show significant variations in transposable element DNA methylation levels (Additional file 2: Figure S2). This result could be explained if *in vivo* siRNA levels are not limiting so that an increase in siRNAs has no clear impact in target methylation levels. Alternatively, *drb2* mutant may accumulate “cytoplasmic only” siRNAs that would be non-functional in methylation. Overall, these results suggest that the loss of DRB2 does not influence the general output of the RdDM pathway (i.e. DNA methylation of targets), at least in standard plant growth conditions, and that the increase in p4-siRNA observed in *drb2* plants is probably the result of a crosstalk with another yet to define pathway.

To learn more about this putative new pathway, we used immunoprecipitation and mass spectrometry to identify DRB2 co-purifying proteins. Most of the significant co-purifying proteins turned out to be epigenetic regulators (see Table 1). Three (out of five tested) DRB2 interacting partners, suggested by mass spectrometry data (HDA19, PRMT4B and MSI4), were confirmed *in vivo* using targeted immunoprecipitation experiments (Figure 2 and Additional file 4: Figure S4) and were found to co-migrate with DRB2 in a 2 MDa complex following gel filtration (Figure 2), suggesting that DRB2 is part of a high molecular weight epigenetic complex. One member of this complex is HDA19, a major plant histone deacetylase involved in a wide variety of gene repressing functions [58-60,78,79]. TPL, a known transcriptional corepressor that interacts *in vivo* with HDA19 to repress root identity genes in the apical part of the embryo [61] and floral identity genes in association with APETALA2 [62], is also present in our data set. Another confirmed member of the complex is PRMT4B, one of the two plant homologues of the animal CARM1 arginine methyltransferase, an enzyme that can mono and dimethylate arginine in position 17 and 26 of histone H3 [80]. PRMT4B, in association with PRMT4A, was shown to repress FLC, but it is not clear at the moment if this repressive effect involves a change in arginine methylation level at this locus [57]. The last confirmed member is MSI4, a substrate receptor of the CUL4-DDB1 E3 ligase that was shown to interact with histone deacetylase (HDA6), with TEK, a transposable element silencing protein, and with members of the polycomb repressive complex 2 to regulate gene expression [69-71]. The nature of these *in vivo* partners suggests that the main function of the DRB2-associated complex is to epigenetically downregulate transcription at targeted loci by inducing a repressive chromatin state. It is intriguing to observe that, in addition to MSI4, three other substrate receptors of the CUL4-DDB1 E3 ligase (PRL1, At3g18060, At2g01330) one CUL4 regulator (CAND1) and one target of the CUL4-DDB1^PRL1^ E3 ligase complex (AKIN10, a Snf1-related protein kinase involved in regulating chromatin remodelling enzymes) are present in our data set [72,73]. Since CUL4-DDB1 complexes have been shown to directly modify histones [81] and to help in the recruitment of enzymes involved in chromatin remodelling or histone modifications [82], it is tempting to propose a central role for these different substrate receptors of CUL4-DDB1 E3 ligase in the organisation and function of the DRB2-containing complex.

One hypothesis to explain the presence of an RNA-binding protein as part of an epigenetic regulator complex is to propose that DRB2 is able to bind structured nascent transcripts thus helping targeting the complex to corresponding transcription sites. Using an RNA immunoprecipitation method we were able to show that DRB2 can indeed bind TE transcripts (but not small RNAs) *in vivo* (Figure 3). This is possibly due to the fact that TE transcripts are likely to contain double-stranded structures and that DRB2 was shown *in vitro* to bind double-stranded RNAs [29]. However, this result does not exclude that DRB2 can bind other type of long structured transcripts *in vivo*. Since one protein associated with DRB2 in the complex is HDA19, the loss of DRB2 could affect targeting of the complex and possibly result in an increase of acetylated histones in *drb2* mutants. Using chromatin immunoprecipitation, we were not able to observe a significant increase in H3K9-K14 acetylation levels in *drb2* plants at the three sites tested (not shown). One possible explanation for this result is the possible functional redundancy between DRB2, DRB3 and DRB5. Out of the five known DRBs, these three share high sequence identity, even outside the boundaries of their DSRMs, and while all simple mutants for these proteins essentially appear wild type, the triple *drb2/drb3/dr5* mutant is severely affected in its growth [80]. Such a severe phenotype would be expected if these proteins all help target a large regulatory complex to chromatin. An alternative, non-exclusive, possibility is that complex recruitment is a multifactorial process, DRB2-binding being only one of different targeting strategies.

## Conclusion

**Figure 4:**
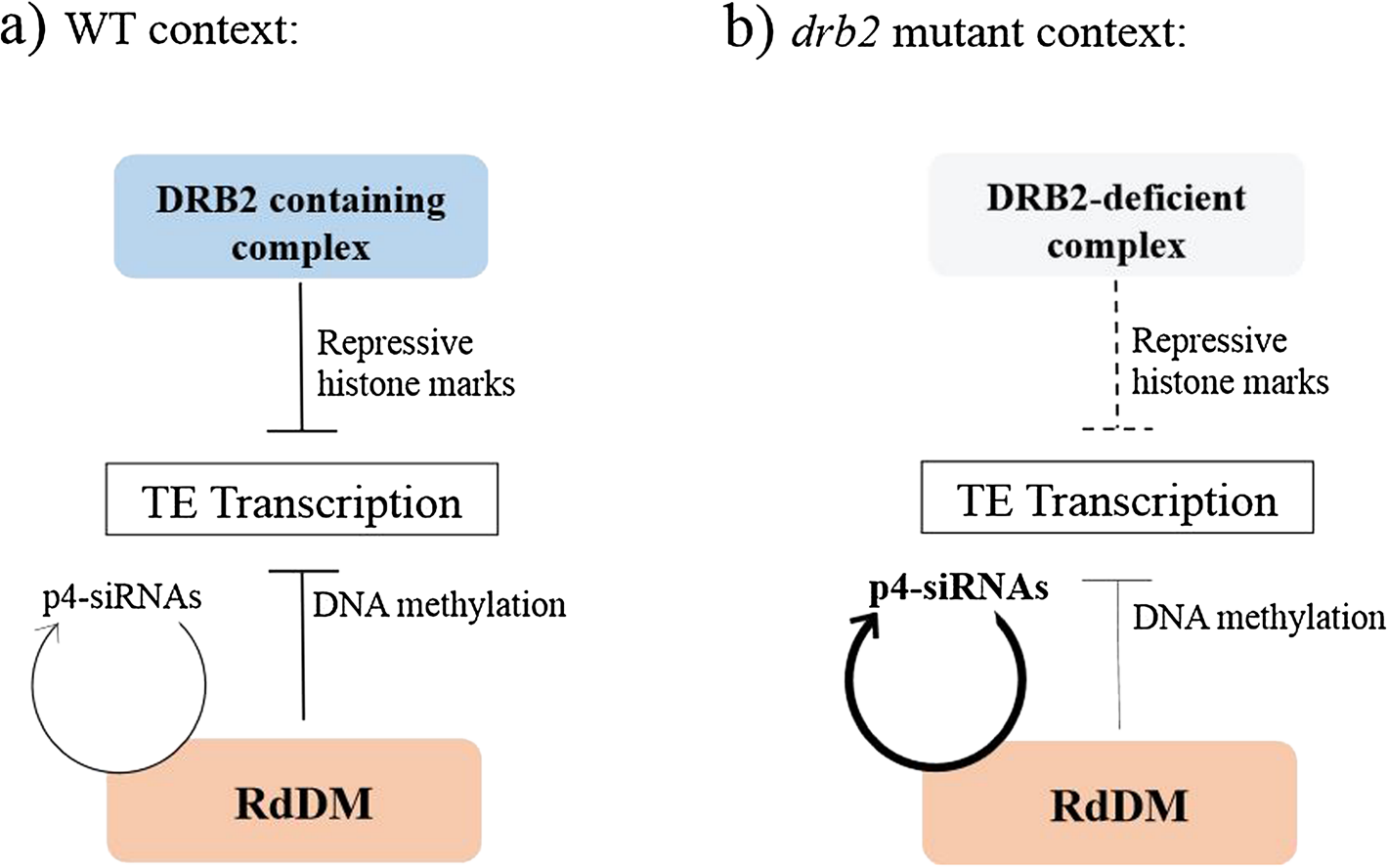
**Proposed model for the action of the DRB2 containing complex, and the resulting situation in the** ***drb2*** **mutant. (a)** In wild type plants, both the RdDM and the DRB2 containing complex act independently to negatively regulate TE transcription. RdDM uses siRNA-mediated DNA methylation to induce silencing while targeting of the DRB2 complex to TE nascent transcript would directly result in an increase in chromatin repressive marks at these loci. **(b)** In a *drb2* plant, targeting efficiency of the complex to nascent transcripts decreases leading to and increase in TE transcription. As no components of the RdDM are impaired, these transcripts are routed to DCL3/RDR2 for p4-siRNA biogenesis leading to the symptomatic over-accumulation of p4-siRNAs observed in the *drb2* mutant without changing the steady state level of TE RNAs.

Based on our results, we propose that DRB2 is involved in targeting a high molecular weight repressive epigenetic complex mainly to TE transcription sites by binding structured nascent transcripts (Figure 4). This complex could operate independently of RdDM in a negative feedback loop to fine-tune transcription, adjusting site by site the epigenetic state to the level of transcripts. According to this model, targeting defects induced in *drb2* mutants would increase transcription at most TE sites, but in the context of a fully functional RdDM pathway, neosynthesized full length TE RNAs would not accumulate to high level but would be converted to siRNAs. Further validation of this model will require a better functional characterization of the DRB2-associated epigenetic complex.

## Methods

### Plant lines and growth conditions

The seed stocks of *drb2-1* (GABI_348A09), *nrpe1-11* (SALK_029919), *ddm1-2* (EMS G to A transition) used in this study are all in the Columbia (Col-0) background and were previously described [83-85]. The NRPD1-Flag, NRPE1-Flag, NERD-FlagHA and HDA19-GFP lines have also been previously described [51-53,86]. Seeds were stratified during at least one day at 4°C before transfer to growth chambers on soil at 23°C under a 16 h-light/8 h-dark regimen. For *in vitro* analysis, seeds were sterilized and sown on Murashige and Skoog (MS) medium including vitamins with 0.8 g.L^−1^ agar, and grown under continuous light at 20°C.

The DRB2-FlagHA, DRB2-Cmyc and PRMT4B-Cmyc constructs were obtained by amplification of the whole genomic region encompassing the promoter and the whole genic sequence minus the STOP codon. This sequence was then fused in C-terminal to either a double Flag double HA or a quadruple Cmyc tag into a pCambia 1300 derived plasmid [52]. Either *drb2-1* or Col-0 plants were transformed with these constructs by floral dipping. Primers used for the cloning strategy are found in the Additional file 6: Table S1.

### RNA isolation and northern blots

Total RNA was extracted from immature inflorescences (stages 1–12) as described in [50]. For small RNA blotting and detection, 10 to 12 μg were heated for 5 minutes at 95°C in 1,5 volume of standard formamide buffer and a constant volumes were loaded into a 15% Acrylamide (19:1 acrylamide:bis acrylamide), 8 M urea, 0,5X TBE gel and separated by electrophoresis. Samples were then electroblotted to Hybond-NX (GE Healthcare) and immobilized following a carbodiimide cross-linking procedure [87]. Hybridization was carried out in 15 ml of ULTRAhyb Buffer (Ambion) overnight at 50°C with a χ^32^P-ATP labelled probe (T4 polynucleotide kinase, Promega, 60 minutes at 37°C). Membranes were washed twice in 3X SSC, 5% SDS and once in 1X SSC, 1% SDS. Oligoprobe sequences are found in [50]. Acquisition and quantification of the signal was achieved with a PMI-FX (BioRAD) phosphoimager and the Quantity One software.

### Protein handling and immunoblot analysis

Protein extracts were obtained by grinding frozen tissues in liquid nitrogen. After resuspension in 2X Laemmli Buffer, the extracts were treated for 5 min at 95°C and centrifuged before loading on SDS/PAGE gels. Samples were electroblotted to PVDF membrane (Immobilon, Millipore) and proteins of interest were visualized using the antibodies described in the text. Antibodies working concentrations were as follow: HA-HRP 1:10000 (H6533 Sigma), Cmyc 1:40000 (sc-789 Santa-Cruz), Flag-HRP 1:7500 (A8592 Sigma), GFP 1:2000 (632592 Clonetech), UGPase 1:10000 (AS05086 Agrisera), H3 1:30000 (07–690 Millipore), DCL1 1:1000, DCL3 1:1000, DCL4 1:500, RDR2 1:5000, AGO4 1:12000. All hybridization were performed in 1X TBS, 0.5% Tween, 5% milk overnight at 4°C.

### Immunoprecipitations

Protein purification of a given epitope tagged protein was achieved with Magnetic Flag-M2 beads (Sigma M8823), Miltenyi magnetic beads and columns (μMACS Cmyc and GFP isolation kit) or Cmyc coupled agarose beads (Sigma A7470). Frozen inflorescences were ground in liquid nitrogen and powder was gently resuspended in 5 volumes of lysis buffer (500 mM Tris pH 8, 150 mM NaCl, 0.1% Igepal, 5 mM MgCl_2_, 10% Glycerol, 1 mM PMSF, 0.25X MG132, 1X Protease inhibitor cocktail (Sigma P9599). The amount of detergent and the nature (NaCl vs KCl) and concentration of salt was adjusted according to specific IP conditions. Crude extracts were allowed to settle during at least 10 minutes on ice, and centrifuged twice at maximum speed for 10 minutes at 4°C. A known volume of crude extract was used to perform binding with an optimal quantity of beads for 30 minutes to 2 hours depending on the kit used, at 4°C with gentle rotation. Beads were then washed two to five times in 1 ml of cold lysis buffer in batch systems, or with 200 μl in the Miltenyi system. Denaturing elution was performed in two volumes of 4X Laemmli buffer or successively with 20 μl and 50 μl of preheated commercial buffer in the case of the Miltenyi columns. Native elution was achieved by competition with 2 volumes of either 250 μg/ml 3x Flag peptide (Sigma F4799) or 500 μg/ml Cmyc peptide (Sigma M2435) for 30 minutes on ice.

For RNA immunoprecipitation, 10 mM Vanadyl Ribonucleoside Complex (VRC, Biolabs S14025S) was added to the lysis buffer, and Flag IP was performed as described with 1 g of mixed floral tissues as starting material. After binding, purified RNAs were directly eluted in 200 μl of guanidium buffer (8MG Guanidinemethylhydrochlorid, 20 mM MES, 20 mM EDTA, pH7) during 10 minutes on ice. Two phenol:chloroform:isoamyl alcool (25:24:1) purification steps were performed and the resulting aqueous phase was precipitated in 2 volumes of absolute ethanol, 20 μg glycogen overnight at −20°C. Pellets were washed in 80% cold ethanol and resuspended in 10 μl of DEPC treated water. 1 μg of the total RNA extracted from the input and 5 μl of the eluted RNAs were DNase treated using the TURBO DNA-*free* Kit (Ambion AM1907) in a final volume of 30 μl. 3U of Turbo DNAse is added for 30 minutes at 37°C twice and is inactivated following the manufacturer’s protocol. 4 μl of treated RNA are used in the reverse transcription reaction (GoScript, Promega A50003) with 0.5 μg random hexanucleotides (Promega C1181) in a final volume of 20 μl, following the manufacturer’s protocol. RT minus controls are obtained by diluting the same volume of RNA in 20 μl of DEPC treated water. 4 μl of cDNA were used in the PCR reaction (GoTaq DNA polymerase, Promega M300) in a final volume of 12.5 μl, and amplified for 37 cycles with the primers found in Additional file 6: Table S1.

### Mass spectrometry analysis

Purified proteins were obtained as described in the immunoprecipitation segment with a starting amount of 1.5 grams of mixed floral tissues. Cmyc IP was performed with 4 Miltenyi columns as described, and elution volumes were pooled and precipitated by addition of 2 volumes of absolute ethanol overnight at 4°C and centrifuged at full speed for 15 minutes. Dry pellets were resupended in 20 μl of 4X Laemmli buffer, denatured for 5 minutes at 95°C and immediately separated by SDS/PAGE. The gel was then fixated overnight in ethanol:acetic acid:water (5:1:4) with gentle shaking, and silver stained using the ProteoSilver kit (PROT-SIL1 Sigma). Bands of interested were cut from the gel and incubated successively in 25 mM ammonium bicarbonate:50% acetonitrile, 25 mM ammonium bicarbonate, ultra-pure water and 100% acetonitrile. Bands were then dried in a speed-vac at room temperature and destained in 7% hydrogen peroxide, washed in 100% acetonitrile and ultra-pure water. Resulting samples were trypsin digested and analysed using a nanoLC-MS/MS LTQ-Orbitrap XL (Thermo Fisher Scientific) in a 40 minutes run/sample. Raw data analysis was performed using MASCOT and further analysis was done using the ‘compare to dataset’ option of Galaxy (https://usegalaxy.org/) to remove unspecific hits that appeared in both the test and control sample. Each peptide was manually checked by BLASTp to obtain a final candidate list, with unique and unambiguous peptides.

### Fractionation

Gel filtration experiments were performed in a Superose 6 10/300 GL column (GE Healthcare) using an ÄKTA-FPLC system. Crude extracts were obtained as described in the immunoprecipitation segment in a buffer without glycerol, centrifuged for 20 minutes at max speed and filtrated on 0.22 μm membranes before injection. 500 μl were injected at an elution speed of 100 μl/minute and fractions of 500 μl were collected. Each fractions was precipitated overnight at 4°C in 2 volumes of absolute ethanol and analysed by western blot.

For isolation of cytosolic and nuclear fractions, mixed floral tissues were resuspended in 5 volumes of 0.5 M hexylene glycol, 20 mM MOPS pH 7, 10 mM MgCl_2_. Crude extract were allowed to settle on ice before filtration on miracloth and 25 μm nylon membrane. Cell lysis was performed by addition of 0.5% TritonX-100 at 4°C with gentle steering for 15 minutes and nuclei were pelleted by centrifugation at 1000 g for 10 minutes. Supernatant containing cytosolic components was further centrifuged at maximum speed for 10 minutes to remove nuclei contaminants while nuclei were sonicated in a Bioruptor UCD-200 with addition of 1% SDS. Resulting samples were divided between the soluble fraction and pellet of the nuclei extract after centrifugation and all the resulting fractions were analysed by western blot.

### Microscopy

Onion cells were bombarded with gold particles coated with a plasmid containing a pDRB2::DRB2genomic-GFP obtained with the same cloning strategy as the other DRB2-tag lines. The bombarded cells were kept twelve hours in the dark and observed by confocal microscopy on a LSM 700 (Zeiss). Twenty-eight cells harbouring GFP signal were observed in two different experiments, all showing the same subcellular localization.

### Supplemental methods

#### Plant lines and antibodies

The DRB2-FlagHA/DRB4-Cmyc double construct was obtained by co-transformation of flower buds with equal quantities of the two binary vectors. Hygromycin resistant progeny was screened for presence of both proteins by western blotting. The DRB1 and DRB5 antibodies were obtained by immunization of rats with epitope peptides from both proteins. Working concentrations were as follows: DRB1 1:1000, DRB5 1:500.

### Bisulfite sequencing

DNA from inflorescences was extracted using the DNeasy plant minikit (Qiagen) following the manufacturer’s protocol. 500 ng of DNA was sodium bisulfite converted using the EpiTech Plus kit (Qiagen) following the manufacturer’s instructions with slight modifications. 2 μl of converted DNA was amplified with a Takara HotStart polymerase (94°C 5 min, followed by 40 cycles of 94°C (45 s), 50°C to 53°C depending on the primers (45 s) and 72°C 1 min, with a final elongation of 10 min at 72°C) with the primers found in Additional file 6: Table S1, and the DNA was purified using the Geneclean turbo kit (MP Biomedicals). 1/10 was cloned into a pGEM-T easy vector (Promega). DNA from colonies was amplified using M13 and M13rev primers and treated with rapidPhosphatase and ExoI at 37°C for 30 minutes. Each clone was then sequenced using the T7 primer. Each clone was aligned to the reference unconverted sequence and incomplete clones were removed from the analysis. Methylation analysis was performed using CyMATE [88].

### DSP crosslinking

DRB2-FlagHA crude extract were obtained by resuspension in 500 mM HEPES pH7.5, 150 mM NaCl, 0.1% Igepal, 5 mM MgCl_2_, 10% Glycerol, 1 mM PMSF, 0.25X MG132, 1X Protease inhibitor cocktail, and treated with 1 mM or 0.5 mM dithiobis[succinimidylpropionate] (DSP 22585, Pierce) during 60 minutes at 4°C with gentle rotation. Constant volumes of the crude extract were removed from the master tube at given time points and treated with 50 mMTris pH 7.5 to stop the reaction. Each tube corresponding to a time point was halved and one half was treated in 4X Laemmli buffer containing 3% β-mercaptoethanol and the other half in 4X buffer without β-mercaptoethanol. Extracts were analysed by western blot.

### Chop PCR

DNA from inflorescences was extracted using the DNeasy plant minikit (Qiagen) following the manufacturer’s protocol. DNA was digested with methylation sensitive HaeIII enzyme (GGCC) and AtSN1/SB4-8, which contains 3 different HaeIII sites was amplified. Disappearance or reduced levels of a fragment after digestion with a given enzyme indicate loss of methylation at that site. RDRP is used as a loading control as it does not harbour any HaeIII sites.

### RT-QPCR experiments

Total RNA was extracted as described above from 3 pools of 2 three weeks old *ddm*1 or *ddm1/drb2* seedlings segregating from a single *ddm1*−/− *drb2*+/− plant. 2 μg of RNA were DNase treated using the Promega RQ1 kit following manufacturer’s recommendations. 0.2 μg of treated RNA was reverse transcribed using PrimeScript RT reagent kit (Perfect real time, Takara) in a final volume of 10 μl using a mix of oligodT and random hexanucleotides as preconized by the manufacturer. One μl of cDNA was used for amplification, using the Eco Real-Time system (Illumina) and SYBR Premix Ex Taq II (Tli RnaseH Plus) (Takara) in a final volume of 15 μl. Each value then represents a mean of three independent biological replicates and standard error of the mean is applied after normalisation to Actin2 gene expression. Primers can be found in Additional file 6: Table S1.

### Coinfiltration of *N. benthamiana* leaves

The *Agrobacteriums* harbouring the MSI4-eGFP and DRB2-FlagHA binary plasmids were grown under the appropriate selection until O.D (600 nm) reached 0.8. 5 ml were pelleted by centrifugation and resuspended in 10 mM MES, 10 mM MgSO_4_, and 100 μM acetosyringone. Equal volumes of each solution was mixed and 5 week old leaves were infiltrated. Plants were maintained in 16 h-light/8 h-dark conditions for 55 hours before IP was performed as described.

### [5′ ^32^P] pCp (cytidine-3′, 5′-bis-phosphate) labelling

Input and eluted RNA from DRB2-FlagHA IPs were labelled with radioactive pCp using the T4 RNA ligase from Ambion (AM2140), following the manufacturer’s protocol. 100 ng of total RNA and half the elution volume were labelled with 3 μl pCp in a final volume of 20 μl overnight at 4°C. RNA was then phenol extracted and precipitated in absolute ethanol, 0.3 M sodium acetate and 20 μg of glycogen. Pellets were washed in 80% ethanol and resuspended in 20 μl of loading buffer (7 M urea, bromophenol blue, 0.5X TBE) and secondary structures were removed by boiling for 5 minutes. Resulting samples were loaded in either 6% or 15% acrylamide (19:1 acrylamide:bis acrylamide) 8 M urea, 0,5X TBE gel and separated by electrophoresis. 6% Acrylamide gels were further dryed for one hour at 80°C under vacuum. Exposure is performed as described in the northern blot section.

## Additional files

Additional file 1: Figure S1. Complementary analysis of the DRB2-FlagHA containing complex. (a) *In vivo* pull-down assays of DRB2-FlagHA with other DRBs. DRB2 does not interact with DRB4-Cmyc, DRB1 and DRB5. Cotransformed plants containing both DRB2-FlagHA and DRB4-Cmyc were obtained to conduct this analysis (left part of the image), while the complemented DRB2-FlagHA line was used for the rest of the analysis (right part of the image). DRB2-FlagHA is revealed with a HA antibody, DRB4-Cmyc is revealed with a Cmyc antibody and DRB1 and DRB5 are revealed using custom made antibodies. (b) Kinetics of the association of the DRB2 containing complex by addition of dithiobis[succinimidylpropionate] (DSP), a cross-linking agent. The DRB2 signal is shifted by addition of 1 mM DSP after 5 minutes and remains as a non-resolved form (>250 kDa) in the SDS-PAGE after up to one hour. The DRB2 signal is shifted by addition of 0.5 mM DSP, with intermediary forms appearing as soon as 1 minute after the start of the assay. In both experiments an important pool of DRB2-FlagHA monomer is observed (between 55 kDa and 70 kDa). Cross-link reversal is controlled by adding βmercaptoethanol to each extract prior to separation by SDS-PAGE. The analysis was performed by western blot and DRB2-FlagHA was revealed with a HA antibody. (c) Gel filtration on a superose 6 column of DRB2-FlagHA crude extracts, fractionated by 250 μl steps. DRB2 is present as soon as the 29^th^ fraction, which is above the maximum peak of dextran blue but still included in the resolving range of the superose 6 column (see silver stained gel). Fractions were analysed by western blot, and DRB2-FlagHA is revealed with @ HA antibody. Fraction numbers, dextran blue elution peak and corresponding volumes are indicated. Additional file 2: Figure S2. DRB2 does not interact with major RdDM component, nor affects methylation status of repeated elements. (a) DRB2-FlagHA does not interact with any of the three DCLs tested. Flag IPs were performed, inputs and purified fractions were analysed by western blot. DRB2-FlagHA is revealed with HA antibody and DCL1, DCL3, DCL4 are revealed using custom made antibodies. (b) DRB2-FlagHA does not interact with RDR2 and AGO4. Flag IPs were performed, inputs and purified fractions were analysed by western blot. DRB2-FlagHA is revealed with HA antibody and RDR2 and AGO4 are revealed using custom made antibodies. DRB2-Cmyc plants were crossed with either NRPD1-Flag plants (c) or NRPE1-Flag plants (d). First generation plants containing both constructs were used to make Cmyc and Flag IPs. Control plants containing only one tagged protein were included as controls. No interaction between DRB2 or NRPD1 and NRPE1 is detected. DRB2-Cmyc is revealed using a Cmyc antibody while NRPD1-Flag and NRPE1-Flag are revealed using a Flag antibody. The *drb2* mutation does not impact the methylation status of SB2-2 (e) and SB3-35 (f). Individual bisulfite converted clones were sequenced and the number (n) is indicated between brackets. CG, CHG and CHH is analysed separately using the CyMATE program. *nrpe1* is included as a control. (g) AtSN1 (SB4-8) SINE copy CHH methylation was analysed by HaeIII digestion (GGCC) followed by PCR (chop-PCR)) in all *drb* mutants. The *nrpe1* mutant is the only one showing CHH hypomethylation (reduced accumulation of the PCR end point PCR product). The RDRP region does not present a HaeIII site and the corresponding PCR product is used as a loading control. Additional file 3: Figure S3. RT Q-PCR analysis of the steady state level of different transposable element RNA in the *ddm1* and *ddm1/drb2* mutant backgrounds. Three pools of two plants each were used for reverse transcription and quantitative PCR. Error bars represent standard deviation from the mean, and the data is presented as a fold change compared to the value obtained in the *ddm1* single mutant. Additional file 4: Figure S4. DRB2-FlagHA and MSI4-eGFP interact in a transient assay. Plasmids containing either both constructs and only the MSI4-eGFP plasmid were agroinfiltrated in *N. Benthamiana*. Leaves were harvested separately 48 hours after inoculation and used to perform a GFP IP. Inputs and IPs were analysed by western blotting. DRB2-FlagHA is revealed with a HA antibody and MSI4-eGFP with a GFP antibody. Additional file 5: Figure S5. (a) Analysis of input and immunoprecipitated RNAs in Col-0 and DRB2-FlagHA by [5′ ^32^P]pCp (cytidine-3′,5′-bis-phosphate) labelling. Labelled RNAs are migrated in a 6% acrylamide gel allowing good resolution of RNA species between 100-nt and 30-nt. No specific signal is observed for DRB2-FlagHA. (b) The same analysis is conducted in a 15% acrylamide gel, allowing for a better separation of small RNAs between 50-nt and 10-nt. No specific signal is observed for DRB2-FlagHA. (c) Biological replicate of the experiment shown in Figure 4. Experiment was performed as described in Figure 4 with the DRB2-FlagHA line, Col-0 and DRB2-FlagHA x *ddm1*. Primers specific to SB2-2 (internal), Evadé and Vandal 21 were used in end point PCR reactions. Additional file 6: Table S1. List of primers used in this study.

## Abbreviations

AGO: ARGONAUTE
CAND: Cullin associated and neddylation dissociated
DCAFs: DDB1-CUL4 associated factors
DCL: Dicer-like
DPS: Dithiobis[Succinimidylpropionate]
DRB: Double-stranded RNA binding proteins
DSRM: Double-stranded RNA binding motif
GFP: Green fluorescent protein
HDA: Histone deacetylase
MSI4: Multicopy suppressor of IRA1 4
NFA: Nucleosome assembly protein
NUC1: Nucleolin-like 1
P4: RNA polymerase IV
PRC: Polycomb repressive complex
PRL1: Pleiotropic regulatory locus 1
PRMT: Protein arginine methyltransferase
RdDM: RNA-directed DNA methylation
RDR: RNA-dependent RNA polymerase
SWI3A: Switch3A
TE: Transposable element
TPL: Topless
