# Supplementary figures and images for: Parallel action of AtDRB2 and RdDM in the control of transposable element expression
Source: BMC Plant Biol. 2015 Mar 3;15:70. doi: 10.1186/s12870-015-0455-z (PMC4351826; doi:10.1186/s12870-015-0455-z)

# Figure S1

a)

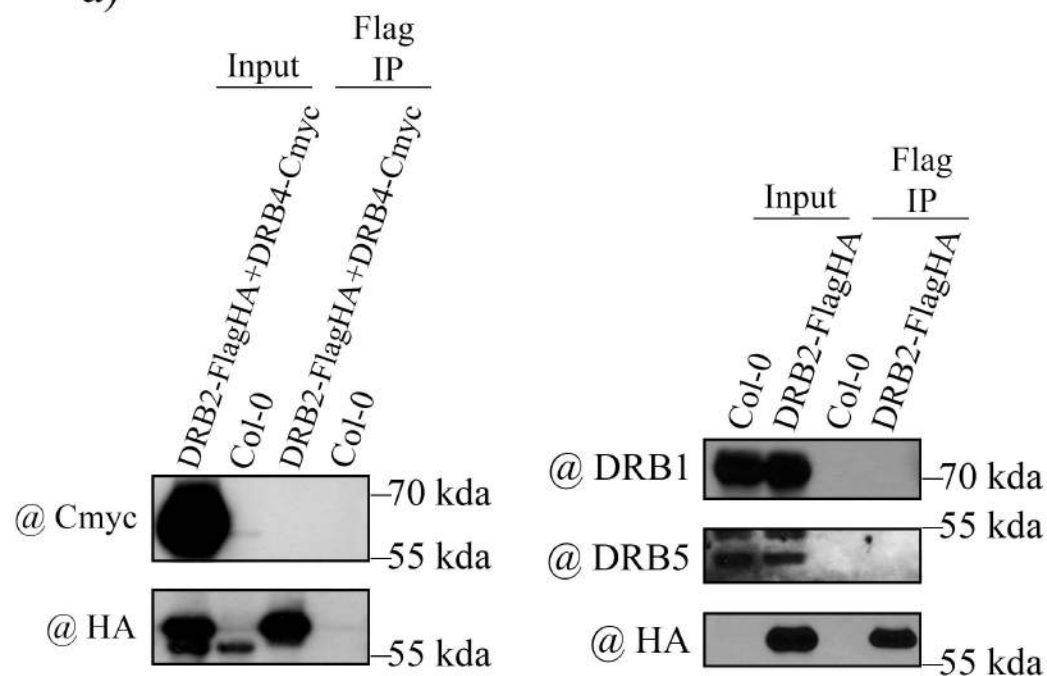

b)

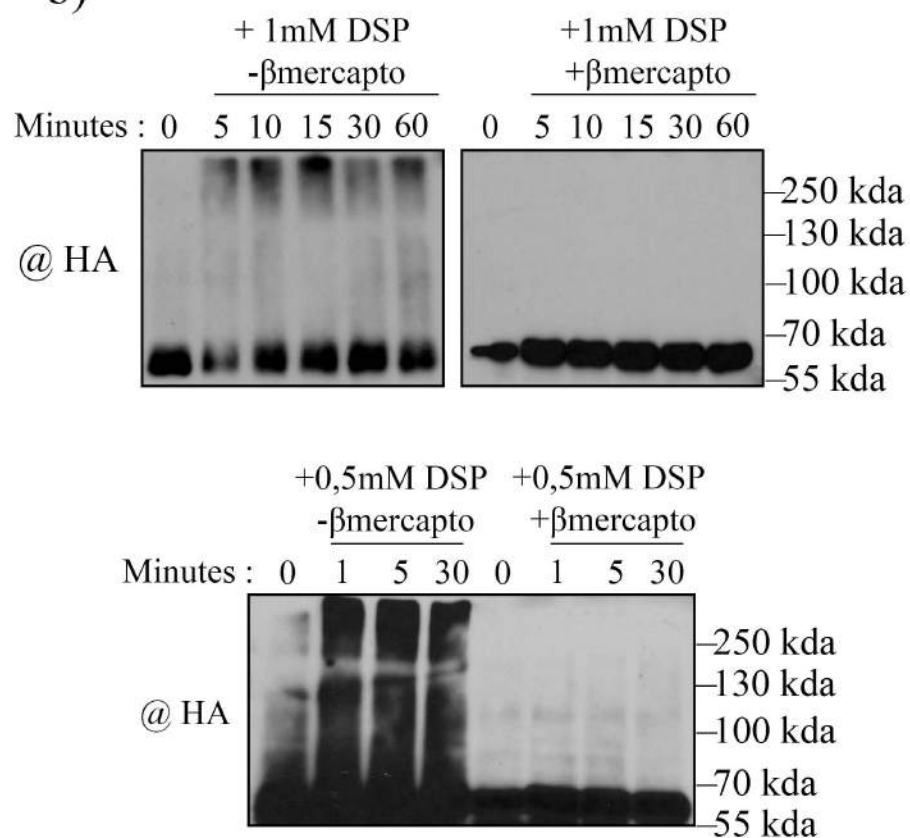

c)

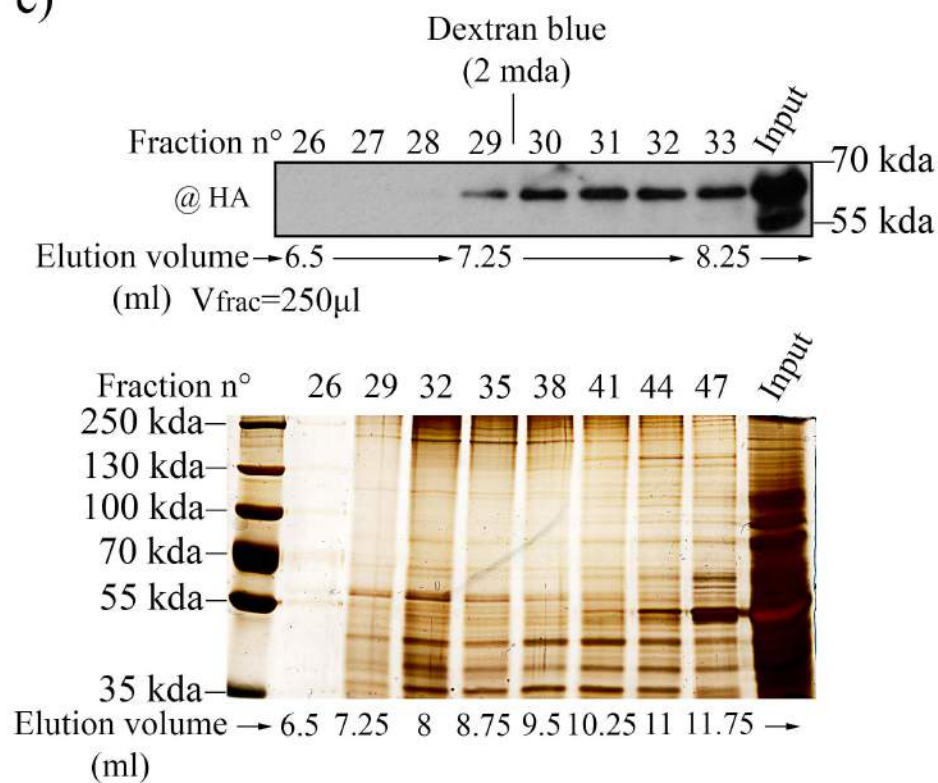

Supplement: Additional file 1: Figure S1. — Complementary analysis of the DRB2-FlagHA containing complex. (a) In vivo pull-down assays of DRB2-FlagHA with other DRBs. DRB2 does not interact with DRB4-Cmyc, DRB1 and DRB5. Cotransformed plants containing both DRB2-FlagHA and DRB4-Cmyc were obtained to conduct this analysis (left part of the image), while the complemented DRB2-FlagHA line was used for the rest of the analysis (right part of the image). DRB2-FlagHA is revealed with a HA antibody, DRB4-Cmyc is revealed with a Cmyc antibody and DRB1 and DRB5 are revealed using custom made antibodies. (b) Kinetics of the association of the DRB2 containing complex by addition of dithiobis[succinimidylpropionate] (DSP), a cross-linking agent. The DRB2 signal is shifted by addition of 1 mM DSP after 5 minutes and remains as a non-resolved form (>250 kDa) in the SDS-PAGE after up to one hour. The DRB2 signal is shifted by addition of 0.5 mM DSP, with intermediary forms appearing as soon as 1 minute after the start of the assay. In both experiments an important pool of DRB2-FlagHA monomer is observed (between 55 kDa and 70 kDa). Cross-link reversal is controlled by adding βmercaptoethanol to each extract prior to separation by SDS-PAGE. The analysis was performed by western blot and DRB2-FlagHA was revealed with a HA antibody. (c) Gel filtration on a superose 6 column of DRB2-FlagHA crude extracts, fractionated by 250 μl steps. DRB2 is present as soon as the 29th fraction, which is above the maximum peak of dextran blue but still included in the resolving range of the superose 6 column (see silver stained gel). Fractions were analysed by western blot, and DRB2-FlagHA is revealed with @ HA antibody. Fraction numbers, dextran blue elution peak and corresponding volumes are indicated. [file 12870_2015_455_MOESM1_ESM.pdf]

Figure S2

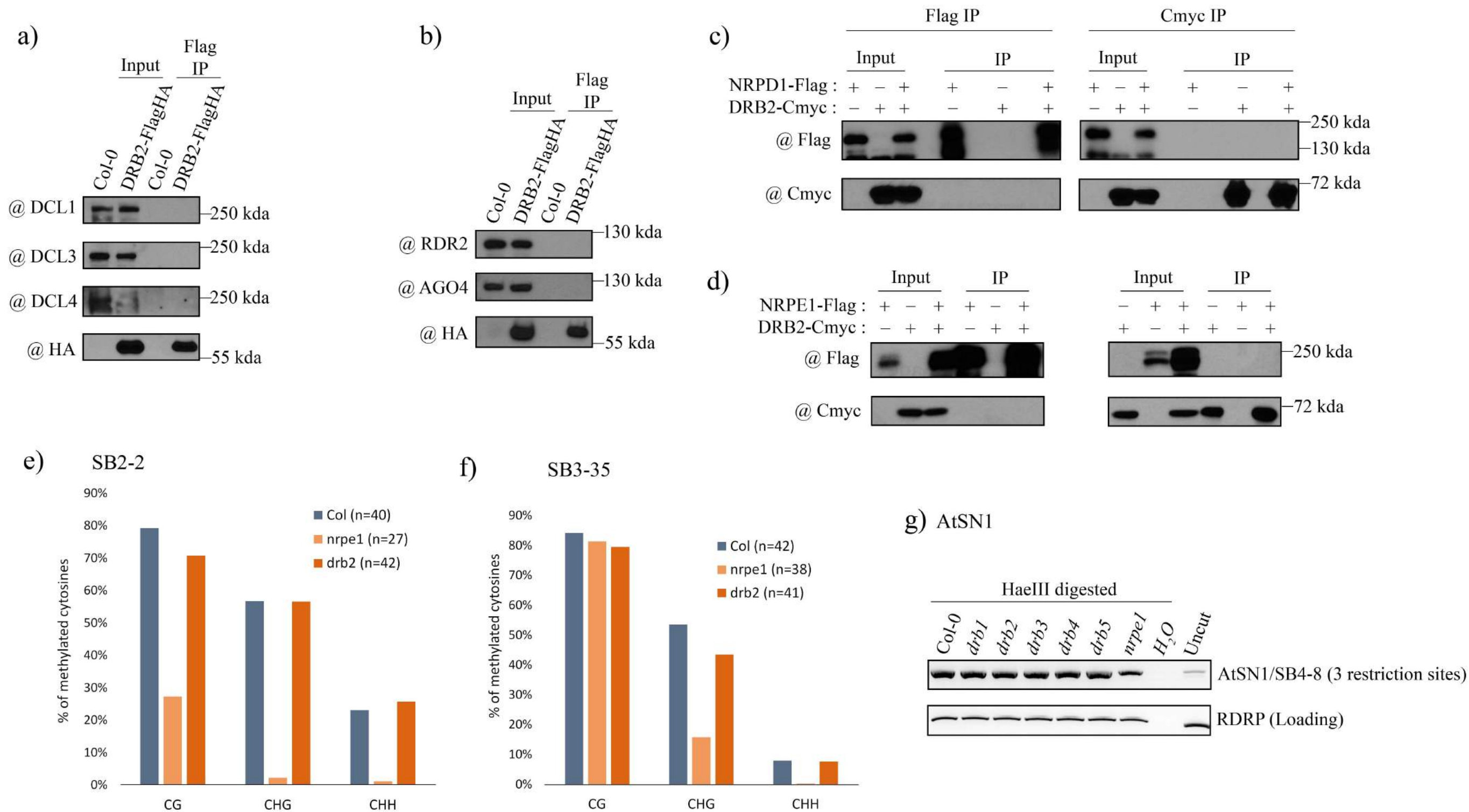

Supplement: Additional file 2: Figure S2. — DRB2 does not interact with major RdDM component, nor affects methylation status of repeated elements. (a) DRB2-FlagHA does not interact with any of the three DCLs tested. Flag IPs were performed, inputs and purified fractions were analysed by western blot. DRB2-FlagHA is revealed with HA antibody and DCL1, DCL3, DCL4 are revealed using custom made antibodies. (b) DRB2-FlagHA does not interact with RDR2 and AGO4. Flag IPs were performed, inputs and purified fractions were analysed by western blot. DRB2-FlagHA is revealed with HA antibody and RDR2 and AGO4 are revealed using custom made antibodies. DRB2-Cmyc plants were crossed with either NRPD1-Flag plants (c) or NRPE1-Flag plants (d). First generation plants containing both constructs were used to make Cmyc and Flag IPs. Control plants containing only one tagged protein were included as controls. No interaction between DRB2 or NRPD1 and NRPE1 is detected. DRB2-Cmyc is revealed using a Cmyc antibody while NRPD1-Flag and NRPE1-Flag are revealed using a Flag antibody. The drb2 mutation does not impact the methylation status of SB2-2 (e) and SB3-35 (f). Individual bisulfite converted clones were sequenced and the number (n) is indicated between brackets. CG, CHG and CHH is analysed separately using the CyMATE program. nrpe1 is included as a control. (g) AtSN1 (SB4-8) SINE copy CHH methylation was analysed by HaeIII digestion (GGCC) followed by PCR (chop-PCR)) in all drb mutants. The nrpe1 mutant is the only one showing CHH hypomethylation (reduced accumulation of the PCR end point PCR product). The RDRP region does not present a HaeIII site and the corresponding PCR product is used as a loading control. [file 12870_2015_455_MOESM2_ESM.pdf]

Figure S3 RT Q-PCR

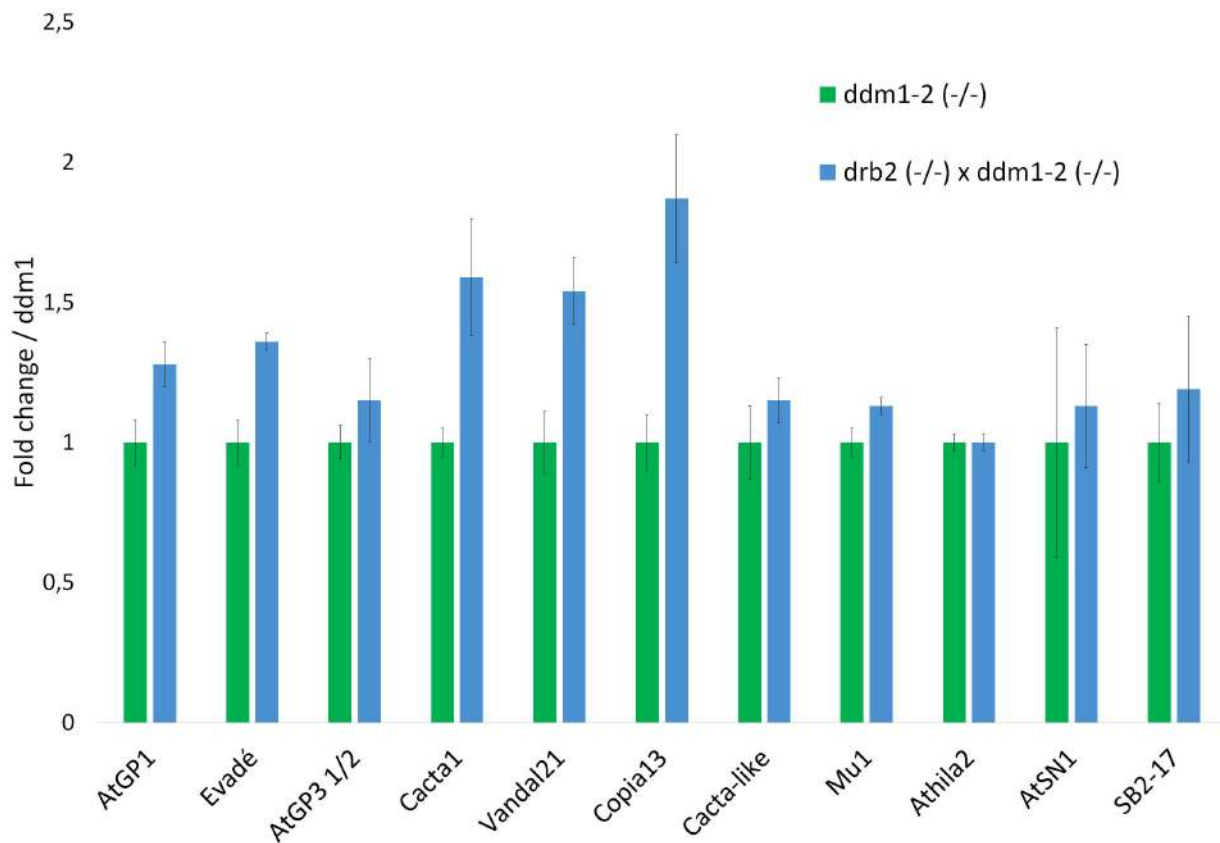

Supplement: Additional file 3: Figure S3. — RT Q-PCR analysis of the steady state level of different transposable element RNA in the ddm1 and ddm1/drb2 mutant backgrounds. Three pools of two plants each were used for reverse transcription and quantitative PCR. Error bars represent standard deviation from the mean, and the data is presented as a fold change compared to the value obtained in the ddm1 single mutant. [file 12870_2015_455_MOESM3_ESM.pdf]

# Figure S4

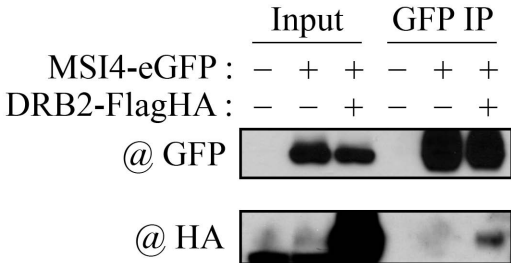

Supplement: Additional file 4: Figure S4. — DRB2-FlagHA and MSI4-eGFP interact in a transient assay. Plasmids containing either both constructs and only the MSI4-eGFP plasmid were agroinfiltrated in N. Benthamiana. Leaves were harvested separately 48 hours after inoculation and used to perform a GFP IP. Inputs and IPs were analysed by western blotting. DRB2-FlagHA is revealed with a HA antibody and MSI4-eGFP with a GFP antibody. [file 12870_2015_455_MOESM4_ESM.pdf]

Figure S5

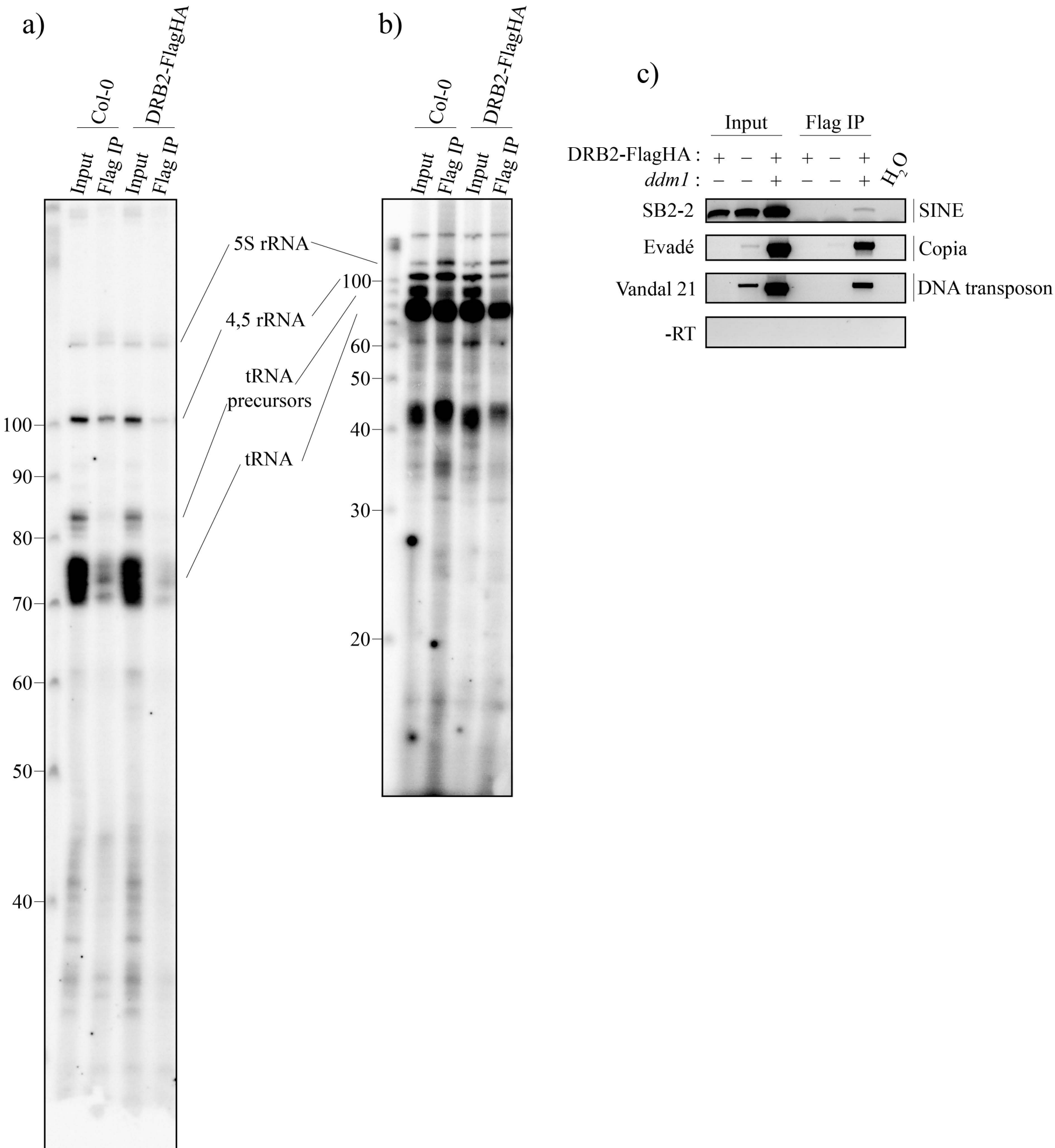

Supplement: Additional file 5: Figure S5. — (a) Analysis of input and immunoprecipitated RNAs in Col-0 and DRB2-FlagHA by [5′ 32P]pCp (cytidine-3′,5′-bis-phosphate) labelling. Labelled RNAs are migrated in a 6% acrylamide gel allowing good resolution of RNA species between 100-nt and 30-nt. No specific signal is observed for DRB2-FlagHA. (b) The same analysis is conducted in a 15% acrylamide gel, allowing for a better separation of small RNAs between 50-nt and 10-nt. No specific signal is observed for DRB2-FlagHA. (c) Biological replicate of the experiment shown in Figure 4. Experiment was performed as described in Figure 4 with the DRB2-FlagHA line, Col-0 and DRB2-FlagHA x ddm1. Primers specific to SB2-2 (internal), Evadé and Vandal 21 were used in end point PCR reactions. [file 12870_2015_455_MOESM5_ESM.pdf]
