# Supplementary material for: Parallel action of AtDRB2 and RdDM in the control of transposable element expression
Source: BMC Plant Biol. 2015 Mar 3;15:70. doi: 10.1186/s12870-015-0455-z (PMC4351826; doi:10.1186/s12870-015-0455-z)
Supplement: Additional file 6: Table S1. — List of primers used in this study. [file 12870_2015_455_MOESM6_ESM.docx]

**Table S1. List of primers used in this study**

| DRB2amp-f | CTATATTGTGCTAGTACCACC | Cloning |
| --- | --- | --- |
| DRB2amp-r | AGACAAAGAGCTTGATTCTGC | Cloning |
| DRB2gclo-f-salI | ATAGATGTCGACTTTGATGGAAGGATGGAGACG | Cloning |
| DRB2gclo-r-pstI | AATCATCTGCAGGATCTTTAGGTTCTCCAGTCG | Cloning |
| PRMT4Bclo-F-XbaI | ATATCTAGACGTTGGACATGATGAAACG | Cloning |
| PRMT4Bclo-R-BamHI | TTAGGATCCGAGCTGGGCACTTGGGTTC | Cloning |
| SB2-17met-dt | AATCAACTCCTTGCAATCTC | RIP |
| SB2-17met-rev | AACATTTATCCATTTCCAATG | RIP |
| E1-17+25 | TAAAGACTCTAGACGAAGC | RIP |
| SB2-2met-dt | ATAAGAGATTTGTTGTATTAATGG | RIP |
| SB2-2met-rev | TGAAAGGTGAGCTCTCTAATC | RIP |
| E1-2 +4 | AAGTGTCGTTAGCTCAATTG | RIP |
| EVD_RT_F | GACCTGACATTTGTTTTGCG | RIP |
| EVD_RT_R | GGTGAAGATATCCGCCAAC | RIP |
| ATGP3_RT_R | CTCTGTAGTTCCTCGGAAAAC | RIP |
| ATGP3_RT_F | CATTAAAATGGTCTGATCGCA | RIP |
| CAC1_I_F | ACTGATAGTGACGATGAATCT | RIP |
| CAC1_I_R | CTGCTGCATCAAGACGTG | RIP |
| VANDAL21_F | GAGTAGAGGAAGAAATGAACG | RIP |
| VANDAL21_R | CGACTTCTTAATCACATAACC | RIP |
| ATHIF6 | CTGTGTGATTTAAGTTTGGG | RIP |
| ATHIR6 | GTTCAAATTGATAGGGAATG | RIP |
| ATHLTRF1 | GCATGTTTAGGATAGTTTTGC | RIP |
| ATHLTRR1 | TCTTCTTTTCTTCCTTGTGCTC | RIP |
| eif1ad | CTAAGGATGGTCAGACCCG | RIP |
| eif1ar | CTTCAGGTATGAAGACACC | RIP |
| LARP6B_F | GGATCCTGAAGGATATGTGCCTATTC | RIP |
| LARP6B_R | GATCCTCTCCTATTGGCTGCTC | RIP |
| DRB4_F | GGTACTCTTAGTGCACTAACTA | RIP |
| DRB4_R | GGATCTCTTGTGGACTGCTCTTC | RIP |
| SB2-2BSP-F | ATTTGTTAGTGTTAATTTATAAGTTTGAAG | methylation analysis |
| SB2-2BSP-R | ATACAAAATAAAAAATAAACTCTCTAATCA | methylation analysis |
| SB3-35BSP-F | AGAGGTAGTGTATAATYGTTAAAAAAGGA | methylation analysis |
| SB3-35BSP-R | TTTTTTTCCRATTAATTTCRATAAA | methylation analysis |
| AtGP1-F | AGTGTGACTGCCAGGAGGAG | q-RTPCR |
| AtGP1-R | CACCCTGTGTCAGCTCAAGA | q-RTPCR |
| AtGP3-F | GGTGCAATGGTTGGAGACTT | q-RTPCR |
| AtGP3-R | AGTTTCTGCGCCTTCACTTC | q-RTPCR |
| evd-F | AATCGAAAGGGGGAGAAAGA | q-RTPCR |
| evd-R | CGCAAAACAAATGTCAGGTC | q-RTPCR |
| cacta1-F | ATTGCATGGCCAGAGGATAA | q-RTPCR |
| SP15 | AACAAAAGCATCATTCTACTTAAC | q-RTPCR |
| Vandal21-F | AGGATGTGCAAGGTGAGTTTCA | q-RTPCR |
| Vandal21-R | ACTCCCGTGATTTCAGCCAA | q-RTPCR |
| AtCopia13-F | TCTCCGTACACATTGGCAAG | q-RTPCR |
| AtCopia13-R | GTCGCCCACTGGTTGTAGTT | q-RTPCR |
| Cacta-like-F | AGCCACTGTCCAGAGCATTT | q-RTPCR |
| Cacta-like-R | AACGATCGACCACTGCTTCT | q-RTPCR |
| AtMu1-F | TCAGAAGGGCTATGGGTGAT | q-RTPCR |
| AtMu1-R | CCCGTGCTTTAATGTCACCT | q-RTPCR |
| Athila2-F | CTCCATTACCCTCACGTGCT | q-RTPCR |
| Athila2-R | GCATCACTACTTCAATCTCG | q-RTPCR |
| AtSN1-F | ATTCGAGACACGTTGGGAAG | q-RTPCR |
| AtSN1-R | TGGTGGTTGTACAAGCCTAGT | q-RTPCR |
| SB2-17-F | TAAAGACTCTAGACGAAGC | q-RTPCR |
| SB2-17-R | AACATTTATCCATTTCCAATG | q-RTPCR |
| Actin2-F | GGCTTAAAAAGCTGGGGTTT | q-RTPCR |
| Actin2-R | TTGTCACACACAAGTGCATCA | q-RTPCR |
| SB2-2 F2 | GCCGGTTCGAGTAATGCT | ChIP Q-PCR |
| SB2-2 R | AATGAAAGGTGAGCTCTCTAATC | ChIP Q-PCR |
| SB2-17 F2 | TAAAGACTCTAGACGAAGC | ChIP Q-PCR |
| SB2-17 R | GTAAAACATTTATCCATTTCCAATG | ChIP Q-PCR |
| TL1738 | ATACCTGAGCGCCCTACTGA | ChIP Q-PCR |
| TL1739 | GGACAAGAAGTGGGAGACCA | ChIP Q-PCR |
